# A Risk Assessment of Antibiotic Pan-Drug-Resistance in the UK: Bayesian Analysis of an Expert Elicitation Study

**DOI:** 10.3390/antibiotics6010009

**Published:** 2017-03-07

**Authors:** Daniel Carter, André Charlett, Stefano Conti, Julie V. Robotham, Alan P. Johnson, David M. Livermore, Tom Fowler, Mike Sharland, Susan Hopkins, Neil Woodford, Philip Burgess, Stephen Dobra

**Affiliations:** 1 National Infection Service, Public Health England, London NW9 5EQ, UK; d-carter@dfid.gov.uk (D.C.); andre.charlett@phe.gov.uk (A.C.); stefano.conti@nhs.net (S.C.); julie.robotham@phe.gov.uk (J.V.R.); tom.fowler@phe.gov.uk (T.F.); neil.woodford@phe.gov.uk (N.W.); 2 Norwich Medical School, University of East Anglia, Norwich NR4 7TJ, UK; d.livermore@uea.ac.uk; 3 Paediatric Infectious Diseases Research Group, St George’s University of London, Cranmer Terrace, London SW17 0RE, UK; mike.sharland@stgeorges.nhs.uk; 4 Antimicrobial Resistance Programme, Public Health England, London NW9 5EQ, UK; susan.hopkins@phe.gov.uk; 5 Health Protection Analytical Team, Department of Health, Richmond House, 79 Whitehall, London SW1A 2NS, UK; philip.burgess@dh.gsi.gov.uk (P.B.); stephen.dobra@btinternet.com (S.D.)

**Keywords:** antibiotic resistance, risk assessment, Bayesian modelling

## Abstract

To inform the UK antimicrobial resistance strategy, a risk assessment was undertaken of the likelihood, over a five-year time-frame, of the emergence and widespread dissemination of pan-drug-resistant (PDR) Gram-negative bacteria that would pose a major public health threat by compromising effective healthcare delivery. Subsequent impact over five- and 20-year time-frames was assessed in terms of morbidity and mortality attributable to PDR Gram-negative bacteraemia. A Bayesian approach, combining available data with expert prior opinion, was used to determine the probability of the emergence, persistence and spread of PDR bacteria. Overall probability was modelled using Monte Carlo simulation. Estimates of impact were also obtained using Bayesian methods. The estimated probability of widespread occurrence of PDR pathogens within five years was 0.2 (95% credibility interval (CrI): 0.07–0.37). Estimated annual numbers of PDR Gram-negative bacteraemias at five and 20 years were 6800 (95% CrI: 400–58,600) and 22,800 (95% CrI: 1500–160,000), respectively; corresponding estimates of excess deaths were 1900 (95% CrI: 0–23,000) and 6400 (95% CrI: 0–64,000). Over 20 years, cumulative estimates indicate 284,000 (95% CrI: 17,000–1,990,000) cases of PDR Gram-negative bacteraemia, leading to an estimated 79,000 (95% CrI: 0–821,000) deaths. This risk assessment reinforces the need for urgent national and international action to tackle antibiotic resistance.

## 1. Introduction

The emergence and spread of antibiotic resistance is a major threat to public health. Infections caused by resistant bacteria are associated with increased morbidity, mortality and economic cost [1]. Many advances in medical care have led, as an unintended consequence, to patients becoming more prone to infection involving opportunistic bacteria. Invasive procedures, immunosuppressive drugs, and the use of intravascular or urinary catheters all compromise the body’s natural barriers to infection. As a result, many patients rely on the therapeutic or prophylactic use of antibiotics to minimize the risk of opportunistic healthcare-associated infections. The widespread occurrence of bacteria that are resistant to antibiotics thus threatens the routine management of patients in diverse clinical settings. In a worst-case setting, the proliferation of strains of bacteria resistant to all routinely available antibiotics, hereafter referred to as “pan-drug-resistant” (PDR) bacteria, would severely compromise many aspects of modern medicine.

The global nature of antimicrobial resistance (AMR) and the need for action have been highlighted in recent reports from Europe [2], the USA [3], Australia [4], and the WHO [5]. In the UK, the threat posed by resistance was emphasized in a report by the Chief Medical Officer [6] and led to the publication of a 5-year national strategy [7]. In support of this strategy, a risk assessment was undertaken of the likelihood of emergence and impact of the widespread occurrence of PDR bacteria, using elicitation of expert opinion [8] coupled with Bayesian statistical inference [9,10].

## 2. Results

### 2.1. Probability of Occurrence

**Figure 1:**
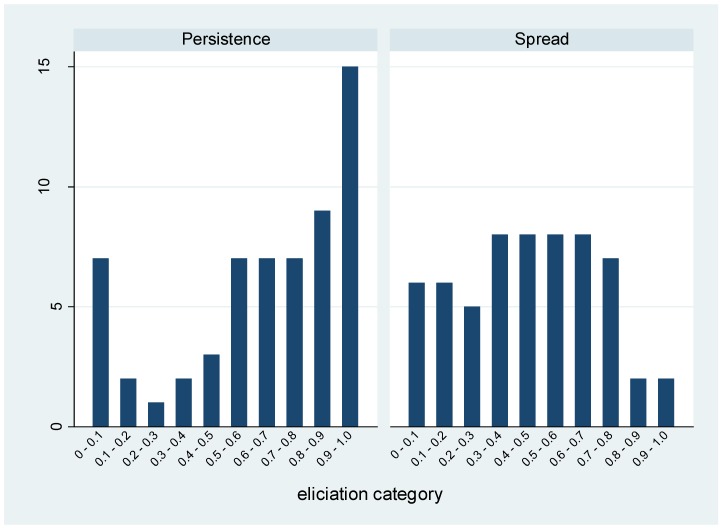
Prior distributions of the expert panel elicitation.

As outlined in Equation (1) (described in detail in the Materials and Methods), the overall likelihood of the scenario was based on the combined probability that PDR bacteria would emerge in the UK, persist and then become widely disseminated. The consensus view of the expert panel was that PDR Gram-negative bacteria had already emerged in the UK, as evidenced by referral of PDR isolates to the national reference laboratory; therefore, the probability of emergence was set to one. The prior opinion from the expert panel on the two remaining terms in Equation (1), together with summary statistics from corresponding prior Beta distributions, are presented in Figure 1.

**Figure 2:**
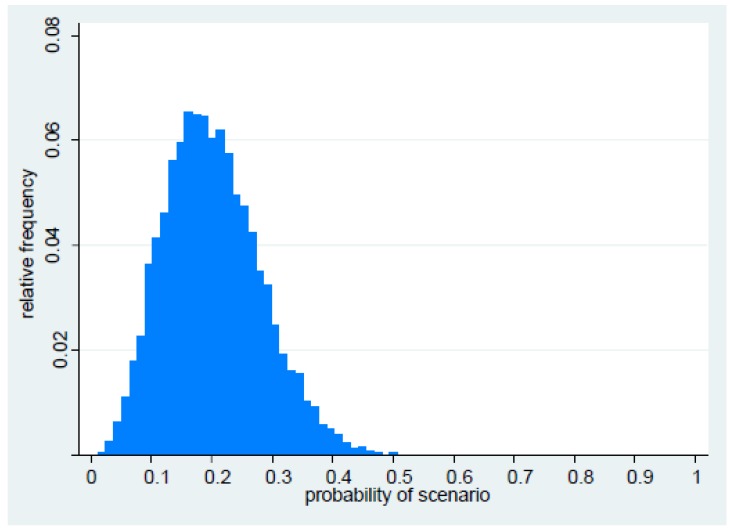
Posterior distribution of the probability of the scenario occurring within five years.

With regard to probability of persistence (given emergence has occurred), analysis of data from the British Society for Antimicrobial Chemotherapy (BSAC) bacteraemia surveillance programme between 2001 and 2012 showed that while emergence of new resistance occurred in four Gram-negative pathogens, only one (carbapenem resistance in *Acinetobacter* spp.) exhibited persistence (Table S3a). In contrast, in 35 instances of pre-existing resistance (inferring persistence), 14 exhibited spread, defined here as a peak annual proportion of resistance of at least 25% (Table S3a). It should be noted that this figure was close to the panel′s elicited estimate of 26% for the peak proportion of PDR. Combining this with the prior distributions in Table 1 yields posterior estimates (95% credibility intervals (CrI)) of 0.50 (0.18–0.82), and 0.40 (0.26–0.56) for the probabilities of persistence and spread, respectively. Taking the data on persistence into account led to a downwards revision of the prior estimate of 0.79 elicited from the panel (Table 1) to a posterior estimate of 0.5, whereas the estimate of spread remained largely unaffected by the data. Figure 2 presents the posterior distribution of the probability on the left-hand side of Equation (1), obtained from Monte Carlo simulation. Based on this, the probability of occurrence of the scenario of PDR bacteria emerging, persisting and spreading as agents of bacteraemia within a 5-year interval was estimated to be 0.19 (95% CrI: 0.07–0.37).

### 2.2. Impact on Patients

The estimated impact of the widespread occurrence of PDR Gram-negative bacteria using a variety of parameters is shown below.

#### 2.2.1. Number of Bacteraemias

Following occurrence of the scenario, the cumulative numbers of PDR Gram-negative bacteraemias in the UK over five- and 20-year periods were predicted to lie between 1100 and 158,000 and 17,000 and 1,989,000, respectively (Table 2), the interval widths reflecting the combined uncertainty from the expert panel’s opinion and the available surveillance data.

#### 2.2.2. Mortality

As with the number of PDR bacteraemias, there was considerable uncertainty in the numbers of attributable deaths. These were cumulatively estimated over five and 20-year periods to be 1900 (95% CrI: 0–23,000) and 6400 (95% CrI: 0–64,000), respectively (Table 3).

#### 2.2.3. Additional Length of Stay

The expert panel members provided lower and upper limits, lower and upper quartiles and a median estimate of the additional length of stay (LoS) following PDR Gram-negative bacteraemia of 8.0, 17.5, 10.5, 14.5 and 13.0 days respectively. Following occurrence of the scenario, the cumulative numbers of additional LoS days over five- and 20-year periods were estimated to be 60,000 (95% CrI: 2600–875,000) and almost 200,000 days (95% CrI: 10,000–2,400,000), respectively (Table 4).

#### 2.2.4. High-Risk Patients

Estimates of the number of PDR infections were made for the groups considered to be at high risk in this scenario. The incidence of surgical site infection (SSI) following large bowel surgery was estimated to rise from 10% in year 0 of the scenario (baseline) to 12% and 18% in years 5 and 20, respectively: it was estimated that about 5000 SSIs from PDR Gram-negative organisms attributable to the scenario would occur over the first five years. For non-elective hip replacement, and repair of fractured neck of femur, it was estimated that about 200 and 400 attributable SSIs involving PDR Gram-negative organisms would occur over the first five years of the scenario.

The impact upon other groups considered the attributable numbers of PDR Gram-negative bacteraemias and so, to some degree, overlapped with previous results. It was estimated that attributable numbers in year 5 of the scenario would be approximately 3700 for patients undergoing flexible cystoscopies, 900 for patients with febrile neutropenia, 900 for renal transplantation, and 90 for renal dialysis patients.

## 3. Discussion

We describe here a quantitative risk assessment that enabled both the likelihood and impact of a challenging, yet realistic, AMR scenario occurring in the UK to be estimated. The risk assessment focused on infections due to PDR Gram-negative bacteria, as this is the clinical setting where the paucity of new or effective old antibiotics is likely to have the most impact. Evidence to inform the risk assessment was gathered by combining data from existing surveillance systems and expert opinion formally elicited from an expert multi-disciplinary panel of healthcare professionals, chosen for their experience and knowledge of the field of antibiotic resistance. A Bayesian statistical analysis was carried out on the collected body of evidence in a manner designed to take account of both variability in the data and epistemic uncertainty in the expert opinion. Estimates of key measures of the healthcare and economic burden to society following the occurrence of the scenario were obtained over both five and twenty-year projected timeframes.

The approach is not new; for example, Kennedy et al. [11] used a similar approach to quantify the risk of Vero cytotoxin-producing *Escherichia coli* O157 infection from milk. However, to our knowledge, it is the first time such an approach has been used to estimate the risk and impact of the advent of a PDR pathogen. Other recent reviews of AMR [12,13] have used a range of scenarios, including those that are unrealistically at the extremes of 0% and 100% resistance across a range of pathogens. While these reviews concentrated on the global economic impact of AMR, the present UK study has a national focus. Furthermore, although those reviews supplemented available data with expert opinion, the uncertainty surrounding it was not taken into account. While all risk assessments are, by their nature, uncertain, in the present study the capturing of all sources of uncertainty, both within the expert panel elicitation and the available data, and their propagation into final estimates through Bayesian modeling, enabled a comprehensive assessment of the uncertainty surrounding the risk analysis. Consequently, we were able to provide an interval estimate for the likelihood of the selected scenario occurring in the next five years of between 0.07 (~1/14) and 0.37 (~1/3), with a median estimate of 0.2 (~1/5). This likelihood is not negligible, and implies a reasonable expectation that persistence and spread of a PDR Gram-negative organism could occur over five years. In the longer term, this equates to an approximate 4% annual chance of the scenario starting in a given year. This in turn suggests that the likelihood over a 20-year period is around 0.8, highlighting the urgent need to take action and mitigate this risk through a range of measures, such as enhanced antibiotic stewardship and development of new generations of antibiotics, vaccines and effective rapid diagnostics.

Although, from a global perspective, surveillance data on AMR may be somewhat sparse, the UK is better served in this respect than many other nations. The likelihood estimate was derived using data from the BSAC Bacteraemia Surveillance Programme, which have been shown to closely mirror other national surveillance data collected from hospital microbiology laboratories around the UK [14]. These are subject to limitations in the sensitivity and specificity of resistance surveillance, and their use also assumes that historical observations of the persistence and spread of resistance are valid predictors in the context of future PDR. The potential for the rapid proliferation of near pan-resistant clones is well illustrated by the expansion of *Klebsiella pneumoniae* carbapenemase (KPC)-producing *K. pneumoniae* of clonal complex (CC) 258. These accounted for most of the early accumulation of carbapenem resistance among *K. pneumoniae* in Italy [15], where the proportion of carbapenem-resistant *K. pneumoniae* rose from 2% in 2008 to 15% in 2010 then to 33% in 2014 [16]. Most representatives of this lineage remain susceptible to gentamicin, polymyxins and tigecycline, though around 16%–22% have acquired resistance to any one of these agents [15]. Although KPC-producing *K. pneumoniae* of CC258 have not yet spread within the UK, despite repeated introduction [17], the pKpQIL plasmids that encode KPC in *K. pneumoniae* of CC258 that have spread widely in Israel [18] are highly related to the plasmids that are spreading among diverse Enterobacteriaceae in north-west England. Additionally, there is growing evidence that KPC carbapenemases are now spreading beyond CC258-related *K. pneumoniae* in Italy, penetrating into a diversity of *Klebsiella* lineages, with an overall colistin resistance rate of 42% [19]. These observations highlight the plausibility of the conclusions reached in the present study.

Point estimates of the cumulative numbers of PDR Gram-negative bacteraemias over the first five and 20 years of the scenario were approximately 20,000 and 280,000, respectively. In the longer term, we estimated approximately 80,000 attributable deaths among the 280,000 cases of bacteraemia. However, the propagation of all the uncertainty in the modeling inputs led to extremely wide credibility intervals around these central estimates.

The estimates of impact necessarily use a number of simplifying assumptions. The projected increase in prevalence of PDR organisms (as a proportion of all Gram-negative bacteraemia isolates) from 0% in year 0 to a peak of approximately 26% in year 20 was determined by modeling the trajectory of increase in prevalence observed with CTX-M extended-spectrum β-lactamases in *E. coli* in the UK and carbapenem-resistant *K. pneumoniae* in Italy and Greece, coupled with expert opinion. An assumption is made that this projected rise in PDR prevalence captures both the spread of pan-resistance and the increased propensity of PDR infections to give rise to bacteraemia as a result of ineffective treatment of underlying infection. The projected baseline number of Gram-negative infections over time, which was required to derive the numbers of PDR Gram-negative bacteraemias from their estimated prevalence, was derived from a simple longitudinal regression model of existing surveillance data.

The numbers of deaths attributable to PDR Gram-negative bacteraemias and of additional hospital LoS days were estimated using data for multidrug-resistant *E. coli* bacteraemias as a proxy for PDR Gram-negative bacteraemias. In particular, estimates of the numbers of attributable deaths will underestimate the true burden of PDR Gram-negative bacteraemias, which is likely to be greater than for multidrug-resistant *E. coli*. Expert elicitation was used to provide a means of assessing this bias and incorporate it into the model. The resulting figures were 1900 (95% CrI: 0–23,000) and 6400 (95% CrI: 0–64,000) over five and 20 years respectively. A lower credibility bound for the 30-day mortality odds ratio of zero might be regarded as implausible, given that the estimated number of PDR cases was never zero. However, this was a reflection of the uncertainty surrounding the opinion elicited from the expert panel on the odds ratio′s *actual* lower bound.

Most of the estimates of incidence of infectious complications of medical procedures were based upon published data (though not all from the UK) applied to Hospital Episode Statistics (HES) from a single year. These estimates should be treated with caution, particularly as no credibility intervals around the central estimates were provided, and the range and frequency of medical procedures performed by the NHS may change over the 5–20-year time-scale considered here.

An estimate of the number of cases of PDR infections at anatomical sites other than the bloodstream was projected using a published estimate of the ratio of Gram-negative bacteraemias to other Gram-negative infections [20]. This ratio of 9% would indicate that the total number of PDR infections may be 10-times greater than our estimates of PDR Gram-negative bacteraemia. However, such extrapolations are highly uncertain: this ratio may change as a result of PDR, particularly if bacteraemias become relatively more common due to ineffective treatment of underlying infections at other body sites.

The findings of this risk assessment indicate that there is a measurable risk of PDR pathogens emerging and becoming endemic in a matter of years. The prospect of widespread untreatable infections reinforces the urgent need for action to mitigate the risk of such an event occurring. Moreover, while the outcomes of this risk assessment were derived from an analysis of data and expert opinion relevant to the UK, the risk to public health posed by AMR is global in nature and other counties may face a similar level of risk. Thus, the response to the threat of AMR needs to be international in scope [21]. To this end, it is encouraging that Heads of State came together to commit to fighting the threat posed by AMR at the UN General Assembly meeting in September 2016 [22].

## 4. Materials and Methods

### 4.1. Expert Panel and Remit

The panel comprised members from academia, the National Health Service (NHS), Public Health England (PHE) and the UK Department of Health (DH), who variously had expertise in antimicrobial resistance, infectious disease epidemiology, clinical microbiology, pharmacy and patient safety. Their remit was three-fold: firstly, to devise a scenario in which the level of antibiotic resistance in the UK made much of modern medicine untenable due to a high prevalence of untreatable infections; secondly, to assess the likelihood of this scenario occurring within a five-year timeframe; and, lastly, to quantify the impact of this challenge over five- and twenty-year horizons.

The panel considered a range of clinical settings, patient populations and pathogens most relevant to the above scenario. The key features envisioned were that a PDR and highly virulent Gram-negative bacterial strain enters or emerges in the UK, resulting in a loss of clinical utility of all available antibiotics. This resistance pattern would rapidly become geographically widespread, through a combination of strain spread along with intra- and inter-species transfer of a promiscuous plasmid encoding both the multi-resistance and virulence traits. Significant mitigation of the outbreak would not be possible due to failure of prevention and control measures to keep pace with the increasing scale of the problem, insufficient effectiveness of rapid diagnostics and unavailability of new agents for effective treatment.

### 4.2. Risk Assessment

A Bayesian analytical approach was used, whereby information elicited from the panel was combined with available data (including unpublished surveillance data). The aim was to estimate the likelihood of such a scenario emerging within five years and to assess its subsequent impact over periods of five and twenty years. Aspects considered included affected patient groups, fatalities, excess morbidity and increased LoS in hospital. Outline methods are presented below, with statistical methodology available as Supplementary material.

### 4.3. Expert Elicitation

During early 2014, opinions on 11 key scenario descriptors (Parameters, Table 5) were formally elicited from expert panel members in the form of probability distributions using the Sheffield Elicitation Framework (SHELF) [23] and the Multidisciplinary Assessment of Technology for Healthcare (MATCH) online elicitation tool [24]. For Parameters 1–5, which are defined as proportions, a “roulette” elicitation method was used, in which each panel member placed ten “chips” amongst as many equally-spaced “bins” spanning the 0 to 1 probability range. For the remaining Parameters, a quartile method was employed, whereby panel members subjectively formulated median, upper and lower quartiles, together with plausible ranges. For each Parameter, distributions elicited from each panel member were thereafter combined into a pooled prior distribution; these distributions were subsequently discussed by all the panel members to reach a consensus prior summarizing the expert panel′s beliefs.

### 4.4. Likelihood of the Scenario

Assessment of the overall likelihood of the scenario was based on the combined probability of three sequential events, as shown in Equation (1), namely the emergence of PDR bacteria in the UK, their persistence and their subsequent widespread dissemination:

*Pr(scenario)* = *Pr(emergence)* × *Pr(persistence/emergence)* × *Pr(spread/emergence, persistence)* (1)

where *Pr(emergence)* is the probability that a PDR organism enters or emerges in the UK at some point within five years and *Pr(persistence/emergence)* is the probability that, following such an event, the organism persists within the UK (i.e., it becomes endemic within a geographical area or setting). Once persistence is established, the final step is spread of the organism (*Pr(spread/emergence, persistence)*) such that it becomes widespread in the population, defined here as a peak annual proportion of resistance of at least 25%. The overall likelihood of the scenario is the product of the likelihood of each of these steps.

Components of the likelihood were considered separately, combining the panel’s prior belief with data obtained from the bacteraemia arm of the BSAC Resistance Surveillance Project [25]. A Monte Carlo simulation approach was adopted to estimate the statistical models for the three above components to finally obtain the overall probability of the proposed scenario.

### 4.5. Impact Assessment

Evaluation of the impact of PDR Gram-negative bacteraemia in terms of morbidity and mortality required two inputs for each year: the projected proportion of Gram-negative infections that were PDR, and the baseline number of Gram-negative bacteraemias. The expert panel′s view was that PDR Gram-negative bacteraemias would independently add to the baseline number of non-PDR Gram-negative bacteraemias. The proportion of PDR cases was informed by Parameters 5 and 6 in Table 5, and the baseline number of Gram-negative bacteraemias derived using Parameter 11. Equation (S3) (see Supplementary Materials) was then used to generate estimates of the number of deaths directly attributable to PDR Gram-negative bacteraemia.

### 4.6. Affected Patient Groups

The impact of PDR infections would be highest in those patients whose vulnerability to infection is increased by aspects of their medical care, such as invasive procedures or immunosuppression. Patient groups included in the risk assessment were therefore those with/undergoing febrile neutropenia, renal dialysis, renal transplantation, flexible cystoscopy, large bowel surgery, hip replacement surgery or repair of fractured neck of femur. Numbers of PDR Gram-negative infections expected in each of these groups were estimated for each year subsequent to the scenario. Projected estimates of the annual number of patients were made using HES [26] for all groups apart from renal dialysis, which used data from the UK Renal Registry [27]. The incidences of Gram-negative infections for large bowel surgery, hip replacement surgery, and repair of fractured neck of femur were obtained from mandatory and voluntary surgical site infection (SSI) surveillance [28]. Incidence estimates from published literature were used for febrile neutropenia [29], renal transplantation [30], and flexible cystoscopy [31]. While no measures of accuracy were calculated, it was recognized that these estimates would exhibit a similar level of uncertainty to that shown by the numbers of PDR Gram-negative bacteraemias.

### 4.7. Hospital Length of Stay

The total additional LoS in days ($S_{i}$) attributable to PDR bacteraemias ($b_{i}$) in year i was calculated using the formula $S_{i}=b_{i}\;\times \;L$ proposed by de Kraker [1], where L is the additional LoS attributable to PDR bacteraemia described by Parameter 9 (Table 5).

## 5. Conclusions

Many medical procedures predispose patients to infection by providing portals of entry for pathogens or by depressing patients’ immune responses. Thus, successful management of patients is frequently dependent of effective antibiotic prophylaxis or treatment. Given the paucity of new antibiotics in development, if resistance to currently available antibiotics becomes widespread, this will adversely impact on delivery of effective medical care in a wide range of clinical settings. This study describes a risk assessment that indicated that there is an approximately 20% chance of such a situation arising in the UK over a five-year time frame. The impact of such an event, were it to occur, would be very significant in clinical and public health terms, with marked increases in morbidity and mortality. This finding reinforces the importance of taking immediate action to tackle the rise in antibiotic resistance.

## Figures and Tables

**Table 1:** Prior distributions for persistence and spread elicited from the expert panel.

Parameter	β Distribution				Percentiles		
	α	β	Mean	Variance	50	2.5	97.5
Persistence	2.96	0.99	0.75	0.04	0.79	0.29	0.99
Spread	1.46	1.72	0.46	0.06	0.45	0.05	0.91

**Table 2:** Point and interval estimates for the annual and cumulative numbers of pan-drug-resistant (PDR) Gram-negative bacteraemia in the UK for selected years of the scenario. CrI, credibility interval.

Year	Median	95% CrI	Median	95% CrI
1	1200	70–7400	1200	70–7400
5	6800	400–58,600	19,600	1100–158,000
10	14,300	800–114,000	77,800	4400–614,000
20	22,800	1500–160,000	283,700	17,000–1,989,000

**Table 3:** Point and interval estimates for the annual and cumulative numbers of deaths attributable to PDR Gram-negative bacteraemia in the UK for selected years of the scenario.

Year of Scenario	Annual		Cumulative	
	Median	95% CrI	Median	95% CrI
1	300	0–3100	300	0–3100
5	1900	0–23,000	5500	0–63,000
10	4100	0–47,000	22,000	0–248,000
20	6400	0–64,000	79,000	0–821,000

**Table 4:** Point and interval estimates for the annual and cumulative additional days length of stay (LoS) attributable to PDR Gram-negative bacteraemia in the UK for selected years of the scenario.

Year of Scenario	Annual		Cumulative	
	Median	95% CrI	Median	95% CrI
1	10,000	500–119,000	10,000	500–119,100
5	60,000	2600–875,000	170,000	8000–2,400,000
10	124,000	5500–1,730,000	676,000	30,000–9,500,000
20	195,000	10,000–2,400,000	2,440,000	120,000–31,900,000

**Table 5:** Parameters elicited from the expert.

Parameter 1: What is the probability that PDR (resulting in loss of susceptibility to all remaining drug classes) in Gram-negative organisms will emerge in or enter the UK within the next five years (i.e., by 2019)?
Parameter 2: In the UK, what proportion of drug class-bug resistance patterns become established, such that they persist over time?
Parameter 3: In the UK, what proportion of established drug class-bug resistance patterns go on to become widespread?
Parameter 4: What is the overall probability that PDR will emerge in or enter the UK within the next five years, and become established and widespread?
Parameter 5: During the scenario, what peak proportion of Gram-negative isolates will demonstrate PDR?
Parameter 6: How many years will elapse from the emergence of PDR, until the peak proportion is reached?
Parameter 7: What cumulative number of PDR Gram-negative bacteraemia will occur during the first five years of the scenario (i.e., 2016–2020)?
Parameter 8: What is the odds ratio for 30-day mortality amongst patients with PDR Gram-negative bacteraemia compared to similar patients with no infection?
Parameter 9: By how many days is length of stay (LoS) greater amongst patients with PDR Gram-negative bacteraemia compared to similar patients with no infection?
Parameter 10: Amongst various potential trajectories for the epidemic curve of PDR Gram-negative bacteraemia (defined in terms of peak prevalence, time to peak prevalence, and the presence or absence of a decline once the peak prevalence is reached), which is considered by the Expert Panel to be the most plausible?
Parameter 11: In addition, panel members were asked to describe the trajectory by which the baseline number of Gram-negative bacteraemias (i.e., non-PDR Gram-negative bacteraemia) may be expected to change over time, to 2035.

## Supplementary Materials

Supplemental material giving detailed statistical methodology is available online at http://www.mdpi.com/2079-6382/6/1/9/s1. The Supplemental material includes: Table S1: Numbers allocated to each elicitation category for the pooled distributions from the expert panel elicitation for parameters denoting proportions, Table S2: Medians of the elicited quantiles from the expert panel elicitation for parameters not denoting proportions, Table S3a: Proportion of resistance in BSAC RSP in antibiotic class-bug combinations between 2001 and 2012 where resistance emerged during this period, Table S3b: Proportion of resistance in BSAC RSP in antibiotic class-bug combinations between 2001 and 2012 where resistant isolate were present in the initial year of surveillance, Table S4: Point and interval estimates for the annual and cumulative numbers of PDR GNB in the UK by year of scenario, Table S5: reports point and interval longitudinal estimates for $d_{i}$, both for individual years and cumulative over time, Table S6: Point and interval estimates for the annual and cumulative numbers of additional LHS in the UK attributable to PDR GNB by year of scenario, Table S7: Point and interval estimates for the annual number of prevalent cases each day in the UK attributable to PDR GNB by year of scenario, Table S8: Estimated number of PDR Gram-negative SSIs (PRGNS) in the high risk patient groups by year.
