# Supplementary material for: A Risk Assessment of Antibiotic Pan-Drug-Resistance in the UK: Bayesian Analysis of an Expert Elicitation Study
Source: Antibiotics (Basel). 2017 Mar 7;6(1):9. doi: 10.3390/antibiotics6010009 (PMC5372989; doi:10.3390/antibiotics6010009)
Supplement: Supplementary file 1 [file antibiotics-06-00009-s001.pdf]

Supplementary Materials: A Risk Assessment of Antibiotic Pan-Drug Resistance in the UK: Bayesian Analysis of an Expert Elicitation Study

Daniel Carter, André Charlett, Stefano Conti, Julie V. Robotham, Alan P. Johnson, David M. Livermore, Tom Fowler, Mike Sharland, Susan Hopkins, Neil Woodford, Philip Burgess and Stephen Dobra on Behalf of the Expert Panel

Expert Elicitation and Bayesian Inference

Elicitation consists of the process of extracting current knowledge and beliefs held by an expert (or pool of experts) about one or more unknown parameters into a (joint) probability distribution for those quantities [1,2]. Elicitation is important in situations in which expert knowledge is essentially the only source of information. It also plays a major role in other contexts by augmenting scarce observational data, through the use of Bayesian statistical methods. O'Hagan et al [3] provide a comprehensive review of the state-of-the-art methods of elicitation and their practical implementation.

Elicitation as an input into statistical modelling falls within the area of subjective Bayesian inference. The Bayesian approach to statistical inference postulates the availability of (i) a prior distribution, conveying knowledge available on any uncertain quantity of interest, and (ii) a likelihood function providing the data-generating mechanism leading to the sample informing those parameters [4]. Bayes Theorem then offers a formally coherent means of revising a prior distribution in the light of the evidence provided by the likelihood function, into a posterior distribution conditional on the collected sample. All inferential statements (e.g., point or interval estimates, hypothesis tests, etc) concerning any parameter of interest follow entirely from its posterior distribution and are usually derived in practice using numerical simulation techniques including (Markov chain) Monte Carlo [5].

Elicited parameters

The pooled raw data collected during the expert elicitation process are summarized in Tables S1 and S2 below.

Table S1. Numbers allocated to each elicitation category for the pooled distributions from the expert panel elicitation for parameters denoting proportions.

**Parameter**	**Elicitation Category**									
(Table 1)	**0·0–0·1**	**0·1–0·2**	**0·2–0·3**	**0·3–0·4**	**0·4–0·5**	**0·5–0·6**	**0·6–0·7**	**0·7–0·8**	**0·8–0·9**	**0·9–1·0**
2	0	0	0	2	3	7	7	7	9	15
3	6	6	5	8	8	8	8	7	2	2
4	17	11	12	11	4	1	1	1	1	1
5	10	12	13	12	4	4	3	2	0	0

Table S2. Medians of the elicited quantiles from the expert panel elicitation for parameters not denoting proportions.

**Parameter**	**Elicitation Category**				
(Table 1)	**Minimum**	**Q1**	**Median**	**Q3**	**Maximum**
6	10·0	15·0	20·0	22·5	25·0
7	750	3750	5500	8000	29,000
8	4·6	6·0	7·0	8·3	9·0
9	8·0	10·5	13·0	14·5	17·5

For parameter 11, the median of the panels elicited baseline number of GNB were 57189, 60458, 66340, 68627, and 70946 for the years 2015, 2020, 2025, 2030 and 2035, respectively.

Risk Assessment Likelihood

The probability of persistence was estimated using a Bayesian approach, combining the consensus expert panel opinion (the prior) assumed to have a flexible Beta distribution, Beta(α, β), with data in the form of a binomial likelihood function (LF) obtained from the BSAC RSP.

The parameters of the Beta priors were estimated from the elicited opinions using the MATCH tool, producing median and 95% uncertainty bounds. The pooled distribution and these parameter values was then presented to and discussed by the expert panel at a workshop, where they felt that the pooled distribution somewhat underestimated the underlying probability. This resulted in a revised median estimate of 0·79 (95% UB: 0·29–0·99). Thus, a Beta (2·96,0·99) distribution was used to express the expert panels prior belief.

Data on persistence, (i.e., the proportion of newly emergent resistance patterns that are sustained over time) were obtained from the BSAC RSP [6] as follows:

a. Data on the number and proportion of bacteraemia isolates in the UK that are resistant to specific antibiotics were extracted, for each of seven organisms of interest (*Acinetobacter* spp., *Citrobacter* spp., *E. coli*, *Enterobacter* spp., *K. pneumoniae*, *P. aeruginosa* and *Serratia* spp.) in each year 2001–2012.

b. Data for individual antibiotics were aggregated by antibiotic class, in order to convert antibiotic-organism combinations into class–organism combinations. This was performed as follows:

b1. Where resistance data were available for only one antibiotic within a class (e.g., gentamicin for the aminoglycosides), that antibiotic was taken to represent the class as a whole.

b2. Where resistance data were available for more than one antibiotic within a class, the overall prevalence of resistance in that class was determined by subtracting from one, the proportion of isolates in which resistance was absent to all of antibiotics within the class.

This resulted in 43 combinations of seven antibiotic classes (penicillins, cephalosporins, aminoglycosides, fluoroquinolones, glycylcyclines, polymyxins, and carbapenems) and seven organisms of interest. No data were available for six class-organism combinations, representing cases where the bacteria are inherently resistant (e.g., Serratia to polymyxins). Emergence events were defined as instances in which resistance was present in any isolate from a given class-organism combination, having been at 0% in all previous years where data were available for that combination (i.e., new detection of resistance). On this basis, resistance emergence events were identified in four class-organism combinations in Table S3a. Of these four events, resistance remained detectable in the BSAC RSP in one of the class-organism combinations, that is carbapenem resistance in Acinetobacter spp. For the remaining three combinations, there were years after the initial year where resistance was first detected where isolates tested exhibited no resistance. Hence, from these four, the estimated proportion of persistence of newly emergent resistance that is sustained over time $\hat{\pi}_p$ is 0·25, with the observed data used in the estimation process following a Binomial (4,0·25) distribution.

The prior and LF were then combined to obtain a posterior distribution which standard probability calculations show to take the form of a Beta distribution, Beta ($\alpha + r, \beta + n - r$) with r denoting the number of successes from the n Binomial trials.

Table S3a. Proportion of resistance in BSAC RSP in antibiotic class-bug combinations between 2001 and 2012 where resistance emerged during this period.

Antibiotic Class-Bug Combinations	Year											
	**2001**	**2002**	**2003**	**2004**	**2005**	**2006**	**2007**	**2008**	**2009**	**2010**	**2011**	**2012**
**Carbapenems**												
Acinetobacter spp.	0·00	0·00	0·00	0·02	0·09	0·13	0·09					
E coli	0·00	0·00	0·00	0·00	0·00	0·02	0·00	0·00	0·00	0·00	0·00	0·00
K pneumoniae	0.00	0·00	0·00	0·01	0·01	0·00	0·01		0·01	0·00	0·01	0·00
Serratia spp.	0.00	0·02	0.02	0·00	0·00	0·04	0·01	0·00	0·00	0·00	0·00	0·00

Table S3b. Proportion of resistance in BSAC RSP in antibiotic class-bug combinations between 2001 and 2012 where resistant isolate were present in the initial year of surveillance.

Antibiotic Class-Bug Combinations	Year											
	2001	2002	2003	2004	2005	2006	2007	2008	2009	2010	2011	2012
**Carbapenems**												
Enterobacter spp.		0·03	0·04	0·04	0·10	0·14	0·10	0·00	0·01	0·02	0·00	0·00
P aeruginosa		0·03		0·03	0·04	0·05	0·05	0·04	0·04	0·05	0·10	0·04
**Aminoglycosides**												
Acinetobacter spp.	0·19	0·04	0·11	0·05	0·33	0·16	0·10					
Citrobacter spp.	0·21	0·00	0·06	0·00	0·00	0·06	0·04					
E coli	0·04	0·04	0·03	0·07	0·07	0·09	0·10	0·06	0·04	0·09	0·08	0·11
Enterobacter spp.		0·10	0·11	0·10	0·13	0·11	0·05	0·07	0·03	0·04	0·06	0·06
K pneumoniae	0·02	0·04	0·08	0·15	0·14	0·13	0·14		0·07	0·09	0·06	0·07
P aeruginosa		0·06		0·08	0·06	0·05	0·04	0·04	0·04	0·02	0·03	0·03
Serratia spp.	0·07	0·05	0·03	0·00	0·03	0·02	0·03	0·00	0·00	0·01	0·00	0·00
**Cephalosporins**												
Citrobacter spp.	0·37	0·23	0·34	0·18	0·15	0·35	0·45					
E coli	0·12	0·12	0·13	0·21	0·25	0·37	0·34	0·36	0·32	0·26	0·32	0·33
Enterobacter spp.		0·34	0·61	0·56	0·52	0·56	0·53	0·44	0·42	0·37	0·34	0·28
K pneumonia	0·04	0·08	0·30	0·50	0·49	0·49	0·47		0·25	0·42	0·30	0·31
P aeruginosa		0·04		0·02	0·04	0·02	0·03	0·03	0·02	0·01	0·02	0·03
Serratia spp.	0·07	0·00	0·36	0·32	0·33	0·30	0·36	0·13	0·16	0·20	0·08	0·10
**Glycylcyclines**												
Enterobacter spp.		0·09	0·02	0·00	0·06	0·08	0·05	0·06	0·04	0·03	0·01	0·02
K pneumonia		0·12	0·01	0·00	0·04	0·05	0·06		0·02	0·04	0·01	0·03
Serratia spp.		0·07	0·00	0·00	0·01	0·05	0·00	0·11	0·02	0·04	0·01	0·02
**Fluoroquinolones**												
Acinetobacter spp.	0·30	0·14	0·16	0·12	0·39	0·29	0·13					
Citrobacter spp.	0·16	0·12	0·06	0·00	0·04	0·06	0·04					
E coli	0·08	0·07	0·11	0·17	0·17	0·26	0·24	0·18	0·14	0·15	0·21	0·22
Enterobacter spp.		0·10	0·09	0·09	0·09	0·11	0·14	0·10	0·07	0·04	0·03	0·04
K pneumonia	0·04	0·10	0·08	0·15	0·18	0·16	0·18		0·08	0·10	0·08	0·11
P aeruginosa		0·11		0·18	0·16	0·13	0·10	0·10	0·04	0·07	0·07	0·06
Serratia spp.	0·14	0·17	0·22	0·25	0·23	0·11	0·14	0·10	0·16	0·11	0·04	0·05
**Polymyxins**												
E coli											0·00	0·01
Enterobacter spp.											0·06	0·06
K pneumoniae											0·01	0·02
**Penicillins**												
Acinetobacter spp.	0·16	0·11	0·24	0·02	0·36	0·26	0·13					
Citrobacter spp.	0·11	0·15	0·13	0·04	0·00	0·16	0·15					
E coli	0·69	0·73	0·74	0·72	0·71	0·82	0·74	0·80	0·77	0·73	0·72	0·76
Enterobacter spp.		0·23	0·26	0·44	0·21	0·18	0·19	0·15	0·14	0·14	0·06	0·06
K pneumonia	0·17	0·18	0·21	0·28	0·26	0·29	0·33		0·20	0·28	0·25	0·20
P aeruginosa		0·04		0·13	0·05	0·02	0·04	0·09	0·05	0·05	0·05	0·05
Serratia spp.	0·14	0·21	0·27	0·30	0·20	0·18	0·21	0·11	0·09	0·16	0·03	0·07

The probability of spread was estimated through an approach analogous to that outlined above, combining the expert opinion prior belief with data obtained from BSAC RSP. The aggregate expert opinion estimate for the median of this parameter was 0.45 (95% UB: 0·05–0·91). No revision was

made to this estimate by the panel at the Workshop. The proportion of resistance patterns within BSAC RSP that underwent spread was estimated from the proportion of antibiotic classes in which resistance was present at the start of the data series, and reached an observed prevalence sufficiently high to deem it to have spread. The data are presented in Table S3b. As there is no single agreed or natural threshold to define spread, the proportion was repeatedly calculated for each of a range of thresholds, at increments of 0·05 (5%), within the interval (0, 0·7). The expert panel's prior opinion was combined with each of these Binomial proportions in turn, in order to generate a range of posterior Beta distributions according to the threshold used to generate the data. The panel supplied an expert opinion for what peak proportion of Gram-negative isolates will demonstrate PR during the scenario, providing a median estimate of 0·26 (95% UB: 0·03–0·69).

The final stage of multiplying the two Beta distributions for the probabilities of sustained resistance and spread was performed using a Monte Carlo procedure. Ten thousand draws yielded an empirical distribution with a mean of 0·20, a variance of 0·006, a median 0·19 and 95% UI of 0·07 to 0·37

Prevalence of PR Bacteraemias

The trajectory by which PDR prevalence would be expected to reach its peak was determined by modelling the trajectories of the increase to peak prevalence observed for ESBL in the UK using BSAC RSP [6], and for MDR *Klebsiella* in Italy and Greece, using EARS-Net data. For each of these outbreaks, the increase in prevalence over time was modelled against a range of growth curve models, with the best fitting function identified via ordinary least squares regression. This information was presented to and agreed by the expert panel. Hence it was determined that the prevalence of pan-resistance p_i would increase linearly from a starting point of 0 in the baseline year 0 to a maximum p^{max} estimated via expert elicitation over 20 years (absolute and interquartile ranges: 10·0–25·0 and 15·0–22·5 respectively).

A Bayesian linear growth model was thus set up to extrapolate p_i for the ith year of the scenario. The model postulates independent exponential prior distributions on the five quantiles elicited by the expert panel on the time to peak PR prevalence, as well as a Beta distribution B(1·5, 3·76) as a prior for p^{max}. The model was fitted via Monte Carlo simulation using the freely available software package JAGS[7] embedded with the R analytical environment [8]. Latin Hypercube Sampling [9] was used to ensure that draws from the distribution of p^{max} were evenly distributed between its four elicited inter-quantic intervals. Linearly extrapolated draws from the distribution of p_i were then combined with simulations from the distribution of yearly baseline (that is non-PR) numbers of GNB b_i^0, whose elicited quantiles were modelled with independent Poisson priors, to derive a Monte Carlo sample from the distribution of PDR GNB b_i for the ith year according to the Equation S1.

$$b_i = \frac{p_i}{(1 - p_i)} . b_i^0 \qquad \text{S1}$$

Baseline Number of GNB

The baseline trend in the number of bacteraemias caused by the Gram-negative organisms of interest was derived from a combination of observed data and expert opinion elicitation. Data on the annual incidence of Gram-negative bacteraemia were obtained from HPA/PHE surveillance reports for England, Wales and Northern Ireland, in order to determine the historic trend in incidence. For each year in which an incidence rate was reported, the rate was applied to the ONS mid-year population estimate for the relevant geographic area, in order to derive an estimate of the number of bacteraemias. Linear regression was applied and used to impute missing years by linear interpolation, to provide a complete set of data on the number of bacteraemias by organism, geographic area and year. Each member of the expert panel then provided their projected (future) trajectory for the total number of bacteraemias to 2040. An upper and lower limit and quartiles for the number of bacteraemias in 2015 and 2035 were additionally elicited, in order to obtain parameters for modelling the uncertainty around projections over time. These trajectories were converted into

numbers of bacteraemias in 2015 and at subsequent five-year intervals to 2040, and the median value in each of those years was proposed to and agreed by the expert panel as its aggregate response. Similarly, the aggregate lower and upper limits and quartiles for 2015 and 2035 were obtained.

The elicited values were then modelled in order to identify the best-fit trajectory of the number of GNB b_i^0 for the ith year over time. The best fitting growth curve model was found to be a quadratic function of time (adjusted $R^2 = 0{\cdot}996$) and is presented in Equation S2.

$$b_i^0 = b_0 + 1213.53y_i - 16.51y_i^2 \quad \text{S2}$$

where b_i^0 is the projected number of bacteraemias in the ith year y_i, and b_0 is the observed number of bacteraemias in year zero (2012).

b_0, the baseline number of bacteraemias in 2012 was obtained from national surveillance data for relevant Gram-negative bacteraemias in England, Wales, Northern Ireland and Scotland. For *E. coli* bacteraemias in England, this was derived from mandatory surveillance. For all other combinations of organisms and geographies, reports originated from voluntary laboratory surveillance. The ratio of voluntarily and mandatorily reported *E. coli* bacteraemias in England (27,367/32,405 = 80%) was used to determine the rate of under-reporting by the former method, and this was in turn used where appropriate to adjust the numbers obtained from voluntarily surveillance. Equation S2 was then used to calculate the number of GNB in each year from 2015 to 2040. Corresponding upper and lower limits and quartiles for each year were derived from the aggregate figures for 2015 and 2035 provided by the expert panel.

Number of PDR GNB

The estimated prevalence of PDR GNB p_i in each year of the scenario was combined with the estimated baseline number of GNB b_i^0 in year(y_i) using equation S1 to obtain estimates of the number of PDR GNB in each year of the scenario, that are presented in Table S4.

Table S4. Point and interval estimates for the annual and cumulative numbers of PDR GNB in the UK by year of scenario.

Year of scenario	Annual Estimates		Cumulative Estimates	
	Median	95% UB	Median	95% UB
1	1200	70–7400	1200	70–7400
2	2400	150–16,900	3600	220–24,000
3	3800	230–29,700	7400	450–54,000
4	5300	300–44,200	12,700	740–99,000
5	6800	390–58,600	19,600	1100–158,000
6	8400	470–71,000	27,900	1600–227,000
7	9900	560–83,700	37,900	2200–311,000
8	11,500	640–94,900	49,600	2800–405,000
9	13,100	720–106,000	63,100	3600–507,000
10	14,300	810–114,000	77,800	4400–614,000
11	15,500	900–120,000	94,000	5300–735,000
12	16,700	970–125,000	112,000	6400–864,000
13	17,900	1100–130,000	130,000	7400–988,000
14	18,700	1100–139,000	149,000	8600–1,124,000
15	19,400	1200–146,000	170,000	9800–1,257,000
16	20,400	1300–144,000	191,000	11,100–1,409,000
17	20,900	1300–150,000	214,000	12,500–1,550,000
18	21,800	1400–150,000	237,000	13,900–1,700,000
19	22,400	1500–155,000	260,000	15,400–1,850,000
20	22,800	1500–160,000	284,000	17,000–1,990,000

Number of Deaths Attributable to PDR GNB

The statistical model for to estimate mortality attributable to PDR GNB proceeds in a similar manner as that outlined for the longitudinal model for PDR GNB. Specifically, independent Normal

priors were assigned to model the minimum, median, quartiles and maximum values for the log-odds ratio (OR) of 30-day mortality elicited from the expert panel. At the same time, a logistic model was utilised for the proportion q of deaths observed from a disease-free sample (148/2180 = 6·79%), obtained from the published literature. Latin Hypercube Sampling ensured uniformity and consistency of Monte Carlo draws from the distribution of log OR with its elicited quantiles. Simulations from the distribution of excess deaths d_i in the ith year attributable to PDR resistance bacteraemiae were obtained according to the relationship in Equation S3 [1].

$$d_i = [b_i.q.(1-q).(OR - 1)]/(q.OR + (1-q)) \quad \text{S3}$$

The median estimates and UB are presented in Table S5.

Table S5. Reports point and interval longitudinal estimates for d_i, both for individual years and cumulative over time.

Year of Scenario	Annual		Cumulative	
	**Median**	**95% UB**	**Median**	**95% UB**
1	300	0–3100	300	0–3100
2	700	0–7000	1000	0–10,100
3	1100	0–11,900	2100	0–22,000
4	1500	0–17,700	3600	0–39,900
5	1900	0–23,500	5500	0–62,600
6	2400	0–28,200	7900	0–92,000
7	2800	0–33,000	10,700	0–124,000
8	3300	0–38,300	14,000	0–160,000
9	3700	0–43,200	17,800	0–203,000
10	4100	0–46,500	22,000	0–248,000
11	4400	0–49,800	26,600	0–299,000
12	4700	0–52,000	31,500	0–354,000
13	5000	0–54,000	36,500	0–414,000
14	5300	0–56,200	42,300	0–464,000
15	5400	0–57,900	48,000	0–524,000
16	5800	0–58,900	54,100	0–582,000
17	5900	0–60,800	60,400	0–642,000
18	6200	0–63,000	66,400	0–702,000
19	6300	0–62,600	72,400	0–761,000
20	6400	0–64,200	79,100	0–821,000

Length of Hospital Stay Attributable to Pan-Resistance

In order to evaluate the impact exerted by sustained spread of PDR GNB over time on health-care resource provision, the expert panel supplied minimum, quartiles and maximum values for the distribution of days of hospital stay LHS_0, assumed fixed over the course of the scenario for simplicity. This was again modelled via Latin Hypercube Sampling in a Monte Carlo framework jointly with the previously reviewed model for the yearly number of PDR bacteraemiae. The additional LHS (in days) attributable to PDR GNB the ith year of the scenario is thus quantified by the relation shown in Equation S4.

$$LHS_i = b_i.LHS_0 \quad \text{S4}$$

Table S6. Point and interval estimates for the annual and cumulative numbers of additional LHS in the UK attributable to PDR GNB by year of scenario.

Year of Scenario	Annual Estimates ('000s)		Cumulative Estimates ('000s)	
	Median	95% UB	Median	95% UB
1	10	0–119	10	0–119
2	21	1–269	31	2–390
3	33	2–458	65	3–843
4	46	2–663	111	5–1520
5	60	3–875	170	8–2410
6	73	3–1082	244	11–3459
7	67	4–1279	332	15–4782
8	100	4–1450	434	20–6321
9	113	5–1606	549	24–7898
10	124	5–1730	676	30–9510
11	135	6–1853	816	36–11,250
12	143	7–1926	971	43–13,333
13	154	7–2070	1126	51–15,417
14	162	8–2080	1291	59–17,318
15	168	8–2170	1469	68–19,376
16	174	9–2283	1654	77–21,748
17	181	9–2315	1843	86–23,949
18	186	9–2422	2044	96–26,696
19	191	10–2400	2241	108–29,005
20	195	10–2406	2440	120–31,904

By using the approximate relationship prevalence ≈ incidence x duration, information on LHS was finally utilised to derive simulation-based inferences on the estimated daily number of inpatients in care in the UK with a PDR GNB (x_i) each year of the scenario using Equation S5.

$$x_i = \frac{b_i}{365.25} \cdot (LHS_0 + LHS_i) \qquad \text{S5}$$

Table S7. Point and interval estimates for the annual number of prevalent cases each day in the UK attributable to PDR GNB by year of scenario.

Year of Scenario	Median	95% UB
1	50	0–400
2	100	10–930
3	150	10–1580
4	210	10–2370
5	280	10–3070
6	340	20–3860
7	400	20–4600
8	470	20–5120
9	520	30–5790
10	580	30–6190
11	630	30–6570
12	670	40–6780
13	720	40–7240
14	750	40–7540
15	780	40–7830
16	810	50–7910
17	850	50–8040
18	870	50–8420
19	890	50–8390
20	910	60–8540

Medical and Surgical Procedures

Patients undergoing large bowel surgery are particularly susceptible to Gram-negative infections. Gram-negative organisms are implicated in a higher proportion of these SSIs than for other categories of surgery for which organism-specific SSI surveillance data are available.

The potential impact of PR Gram-negative infections upon large bowel surgery may be illustrated as follows: In 2008/9–2012/13, 16,734 large bowel surgery procedures were reported to the SSI surveillance programme, amongst which 1772 SSIs occurred. Hence the proportion of procedures in which SSI occurred is 1772/16,734 = 0·106 (95% confidence interval: 0·101–0·111). According to data from the SSI surveillance programme, in 2012/13 Gram-negative organisms were implicated in 103 out of 170 SSIs of known microbiological aetiology. Hence the proportion of SSIs in which a Gram-negative organism is implicated is 0.606 (95% confidence interval: 0·528%–0·680%).

HES reports that 56,846 large bowel surgical procedures were performed in England in 2011/12. Extrapolating by population size, this equates to 67,697 procedures for the UK as a whole. On this basis, and using the point estimates set out above, it may be estimated that there were 67,697 x 0·106 x 0·606 = 4343 large bowel surgery Gram-negative SSIs in the UK in 2011/12. Hence the odds of Gram-negative SSI following large bowel surgery are 4343/(67,697–4343) = 0·07.

Surgical antibiotic prophylaxis (SAP) is widely used for large bowel surgery, and so the odds of Gram-negative infection are not representative of the underlying force of infection in large bowel surgery. In large bowel surgery, the odds ratio of SSI with SAP is 0.24 (95% Confidence Interval 0·13–0·43). On the basis of this point estimate, the underlying odds of SSI (in the absence of SAP) would be 0·07/0·24 = 0·29. As a result, in the absence of SAP one would expect 67,697 x 0·29/1·29 = 15,041 Gram-negative SSIs to occur. The number of procedures performed in England in 2011/12 previously obtained from HES (8.3) was expanded pro-rata by population to derive an estimated number of procedures performed in the UK as a whole. The age distribution of large bowel procedures obtained from SSI surveillance data was applied to the total estimated number of procedures performed in the UK in order to derive estimated numbers of procedures by age-group. These numbers of procedures were then divided by the UK population in each age group, in order to derive age-group specific rates. These rates were then applied to the age-group specific population projections in each year, and the numbers were summed within each year in order to derive a total projected number of procedures by year.

It is projected that in 2019 75,056 large bowel procedures will be performed in the UK. At this number of procedures and based on the odds previously calculated, the underlying force of infection would equate to (75,056 x 0·29)/1·29 = 16,676 Gram-negative infections.

Using the median estimate of 9·4% for the projected prevalence of PDR in year five of the scenario, and the previous assumption that PDR will add to the existing burden of infection, the force of PDR infection would equate to (16,676 x 0.094)/(1-0.094) = 1739 infections. Antibiotic prophylaxis would not be effective in preventing these infections, because they are not susceptible to antibiotics.

Table S8. Estimated number of PDR Gram-negative SSIs (PRGNS) in the high-risk patient groups by year.

Year of Scenario	Non-Elective Hip Replacement Surgery		Repair of Fractured Neck of Femur Surgery		Large Bowel Surgery	
	Procedures	PRGNS	Procedures	PRGNS	Procedures	PRGNS
1	101,000	25	56,000	10	71,000	300
2	102,000	50	58,000	20	72,000	630
3	103,000	75	59,000	40	73,000	980
4	104,000	100	61,000	50	74,000	1350
5	104,000	130	62,000	70	75,000	1740
6	105,000	160	65,000	80	76,000	2130
7	106,000	190	67,000	100	78,000	2540
8	106,000	220	69,000	120	78,000	2930
9	107,000	250	71,000	140	79,000	3300
10	107,000	270	73,000	160	80,000	3670
11	108,000	290	74,000	170	81,000	3960
12	109,000	310	76,000	180	82,000	4240
13	109,000	330	79,000	200	84,000	4520
14	110,000	340	81,000	210	85,000	4800
15	110,000	360	84,000	230	86,000	5000
16	111,000	370	86,000	240	87,000	5240
17	112,000	380	90,000	260	88,000	5470
18	112,000	390	93,000	270	89,000	5640
19	113,000	400	96,000	290	90,000	5800
20	113,000	410	98,000	300	91,000	5950

Also under the previous assumption that PDR adds to the existing burden of infection, these PR infections would arise in addition to the number of infections that would anyway have occurred in the absence of PDR. This latter number of infections may be estimated as follows: 75,056 procedures x 0·106 = 7,948 SSIs, including 7948 x 0.606 = 4815 Gram-negative SSIs which are not PDR. Hence in total 9687 SSIs would occur, of which 1739 would be caused by PDR Gram-negative organisms, 4815 by Gram-negative organisms that are not PDR, and 6555 by organisms other than Gram-negatives. If 7,948 SSIs would have occurred in the absence of PDR, then the estimated additional burden of PR Gram-negative SSIs would represent a 9687/7948 = 1·22 = 22% increase in the incidence of SSIs following large bowel surgery. This analysis was repeated for each year of the scenario and for orthopaedic surgical procedures, and results presented in Table S8.

For other medical and surgical procedures in which Gram-negative infection is particularly important, the existing burden of infection and the potential impact of PDR may be illustrated as follows:

Oncology (febrile neutropenia)

In 2012/13, there were 27,486 episodes of agranulocytosis (D70) recorded in HES data, in which there was also a mention of sepsis (A40, A41), and of either malignancy (C00 - C98) or anti-cancer drugs causing adverse effects (Y43·1 - Y43·3). Extrapolating by population, this equates to 32,733 such episodes per year in the UK as a whole.

According to one study, 89 out of 253 patients with febrile neutropenia had detectable bacteraemia, and in 19 of these the organism was identified as a Gram-negative. One this basis, one might expect to see 32,733 x (89/253) x (19/89) = 2458 episodes of detectable Gram-negative bacteraemia amongst patients with febrile neutropenia in the UK each year. If the magnitude of impact of PR on febrile neutropenia is similar to that projected for large bowel surgery, the number of Gram-negative bacteraemias might increase to 3346 (an additional 888 infections) at a prevalence of PR of 0·094 projected to occur in year 5 of the scenario.

Dialysis

Over an eleven-month period in 2011/12, 191 cases of *E. coli* bacteraemia amongst patients in

England receiving dialysis for established renal failure were reported to the Renal Registry. Extrapolating by population and to 12 months, this equates to 248 cases of *E. coli* bacteraemia per year in the UK as a whole.

If the impact of PDR on dialysis is similar to that projected for large bowel surgery, this number of *E. coli* bacteraemias might increase to 338 (an additional 90 cases) at a prevalence of PDR of 0·094 (projected to occur in year 5 of the scenario).

Transplant surgery

In 2012/13, 2454 kidney transplants were performed in England. Extrapolating by population, this equates to 3031 kidney transplants in the UK as a whole. In one study, 284 infectious episodes occurred amongst 192 kidney transplant patients, each followed up for one year following transplantation. 88% of these episodes were due to bacterial infection, and of these 63% were identified as Gram-negative organisms. Hence the incidence of Gram-negative infection was 284/192 x 88% x 63% = 0·82 episodes per patient year. On this basis, one would expect to see 3031 x 0·82 = 2472 episodes of Gram-negative infection amongst new recipients of kidney transplants in the UK each year. If the impact of PDR on kidney transplantation is similar to that projected for large bowel surgery, this number of infections might increase to 3365 (an additional 893 infections) at a prevalence of PDR of 0·094 projected to occur in year 5 of the scenario.

Urological surgery

In 2012/13, 256,433 flexible cystoscopy procedures were performed in England. Extrapolating by population, this equates to 305,383 flexible cystoscopies in the UK as a whole. In one study, five out of 103 patients developed significant bacteriuria within 48 hours of undergoing flexible cystoscopy, and in six out of nine patients with bacteriuria who had undergone flexible cystoscopy or urodynamic studies, the organism was Gram-negative (E coli). Hence the incidence of Gram-negative bacteriuria within 48 hours of flexible cystoscopy was (5/103) x (6/9) = 0·03.
On this basis, one would expect to see 305,383 x 0·03 = 9883 episodes of Gram-negative bacteriuria within 48 hours of flexible cystoscopy in the UK each year. If the impact of PDR on flexible cystoscopy is similar to that projected for large bowel surgery, this number of infections might increase to 13,453 (an additional 3569 infections) at a prevalence of PDR of 0·094 (projected to occur in year 5 of the scenario).

References

1. Kadane, J.B.; Wolfson, L.J. Experiences in Elicitation. Journal of the Royal Statistical Society: Series D (The Statistician) **1998**, doi:10.1111/1467-9884.00113.
2. Garthwaite, P.H.; Kadane, J.B.; O'Hagan, A. Statistical Methods for Eliciting Probability Distributions. Journal of the American Statistical Association **2005**, doi: 10.1198/016214505000000105.
3. O'Hagan, A.; Buck, C.E.; Daneshkhah, A.; et al. Uncertain Judgements: Eliciting Experts' Probabilities. Chichester: John Wiley & Sons (Statistics in Practice), 2006:,xi+321.
4. Bernardo, J.M.; Smith, A.F.M. Bayesian Theory (2nd edition). Chicester: John Wiley & Sons (Wiley Series in Probability and Statistics), **2000**, doi: 10.1002/9780470316870
5. Gelman, A.; Carlin, J.B.; Stern, H.S.; Dunson, D.B.; Vehtari, A.; Rubin, D.B. Bayesian Data Analysis (3rd edition). Boca Raton: Chapman \& Hall/CRC (Texts in Statistical Science), 2014: xiv+639.
6. British Society for Antimicrobial Chemotherapy Resistance Surveillance Project Avaliable online: http://www.bsacsurv.org/index.jsp?menutype=Main Menu&submenu=Y&parent=Home (accessed on 5 March 2014).
7. Plummer, M. JAGS: A Program for Analysis of Bayesian Graphical Models Using Gibbs Sampling. Proceedings of the 3rd International Workshop on Distributed Statistical Computing. March 2003; 2003; 20–22.
8. R Core Team. R: A language and environment for statistical computing. Vienna: R Foundation for Statistical Computing, 2015. Avaliable online: https://www.R-project.org/ (accessed on 19 November 2015).

9. McKay, M.D.; Beckman, R.J.; Conover, W.J. A Comparison of Three Methods for Selecting Values of Input Variables in the Analysis of Output from a Computer Code. *Technometrics* **1979**, doi:10.2307/1268522.

© 2017 by the authors; licensee MDPI, Basel, Switzerland. This article is an open access article distributed under the terms and conditions of the Creative Commons by Attribution (CC-BY) license (http://creativecommons.org/licenses/by/4.0/).
